# Elevated cardiometabolic risk markers in evening chronotype shift workers: a case–control study in male workers

**DOI:** 10.1017/S000711452510545X

**Published:** 2025-12-14

**Authors:** Amanda S. Wanigasinghe, Dilki S. Perera, Kumari M. Rathnayake

**Affiliations:** Department of Nutrition and Dietetics, Faculty of Livestock, Fisheries and Nutrition, Wayamba University of Sri Lanka, Makandura 60170, Sri Lanka

**Keywords:** Cardiometabolic risk factors, Chronobiology, Chrono-nutrition, Chronotype, Circadian misalignment

## Abstract

Shift work-induced circadian disruption has been linked to various cardiometabolic diseases, including obesity, diabetes and CVD. Limited studies have explored the impact of different variables such as night work durations, intensities and chronotype on cardiometabolic risk. This study aimed to determine the impact of circadian disruption on cardiometabolic risk markers in shift workers. This case–control study was conducted with 104 male workers (shift workers; *n* 52, mean age; 43·3 (sd 10·2), and non-shift workers; *n* 52, mean age; 41·2 (sd 9·8)). Shift work details were determined via an interviewer-administered questionnaire. Cardiometabolic risk was evaluated through anthropometric (height, weight, waist circumference and body composition), biochemical (fasting glucose and lipid profile), clinical (blood pressure) and dietary assessment (24-h recalls from working and non-working days). The chronotype was determined via the Munich Chronotype Questionnaire (MCTQ). Shift workers had significantly higher mean body fat percentage (31·7, 22·7 % *P* = 0·031), systolic blood pressure (SBP) (138·6, 128·5 mmHg *P* = 0·009), pulse rate (78·7, 72·3 bpm *P* = 0·015), TAG (1·60, 1·30 mmol/l *P* = 0·021) and LDL-cholesterol (3·90, 3·40 mmol/l *P* = 0·012) than non-shift workers. Evening chronotype shift workers had significantly higher visceral fat levels (12·8, 8·90 *P* = 0·001), SBP (137·0, 127·6 mmHg *P* = 0·006), pulse rate (82·7, 73·3 bpm *P* = 0·005) and LDL-cholesterol (4·00, 3·40 mmol/l *P* = 0·039) than shift workers with a morning chronotype. In conclusion, shift workers exhibited higher metabolic risk markers than non-shift workers. Shift workers with evening chronotypes had higher cardiometabolic risk than morning chronotypes. Further research is warranted to elucidate the underlying mechanisms and inform targeted interventions for individuals engaged in shift work, considering chronotypes.

Circadian rhythms play crucial roles in governing the physiological and behavioural functions of the human body. Numerous observational studies have established a connection between circadian disruption and the onset of cardiometabolic diseases^(1,2)^. Circadian disruption can result from various lifestyle and environmental factors. Multiple factors, including shift work, late chronotype, late sleep timing, sleep irregularity and late meal timing, have been identified as disruptors of circadian rhythm alignment^(3)^. These factors are associated with potential adverse effects on cardiometabolic health, such as increased BMI/obesity, increased blood pressure, increased dyslipidemia, inflammation and diabetes^(1)^.

Shift work is a risk factor for conditions like overweight, obesity, type 2 diabetes, increased blood pressure and metabolic syndrome. It also influences eating behaviour, food choices, energy intake and macronutrient consumption^(2)^. Night-shift work, in particular, not only leads to a misalignment between the body’s internal circadian system and the external light–dark cycle but also induces internal desynchronisation among various levels of the circadian system and disrupts the expression of clock genes in various tissues. Metabolomics studies revealed shifts in metabolite timing during night work, further misaligning with the circadian system^(4)^.

Chronotype has been shown to play a role in the effect of shift work on health^(5)^. To date, multiple studies have reported that morning types may be less able to adapt to shift work than evening types^(5–7)^. The chronotype is hypothesised to influence the relationship linking shift-induced circadian disruption to cardiometabolic outcomes^(8,9)^. It was observed that individuals with an evening chronotype presented elevated levels of proteins previously associated with cardiometabolic risk^(10)^. Few studies have explored this connection, and the findings are inconclusive. Some studies propose that both morning-oriented and evening-oriented chronotypes could contribute to the risk of cardiometabolic outcomes^(11,12)^. However, research into the role of chronotype in the effect of shift work on metabolic risk factors is still lacking^(13)^.

The influence of circadian misalignment on cardiometabolic health has been extensively studied, but there exists a notable gap in understanding how these dynamics manifest among adults specially security officers, particularly those engaged in night-shift work. Therefore, the aim of our present study was to determine the impact of the chrono effect on cardiometabolic risk markers in shift workers using night work parameters and chronotype as key variables. This case–control study would be beneficial in designing effective interventions to prevent cardiometabolic diseases among shift workers.

## Methods

### Study design and participants

**Figure 1.**
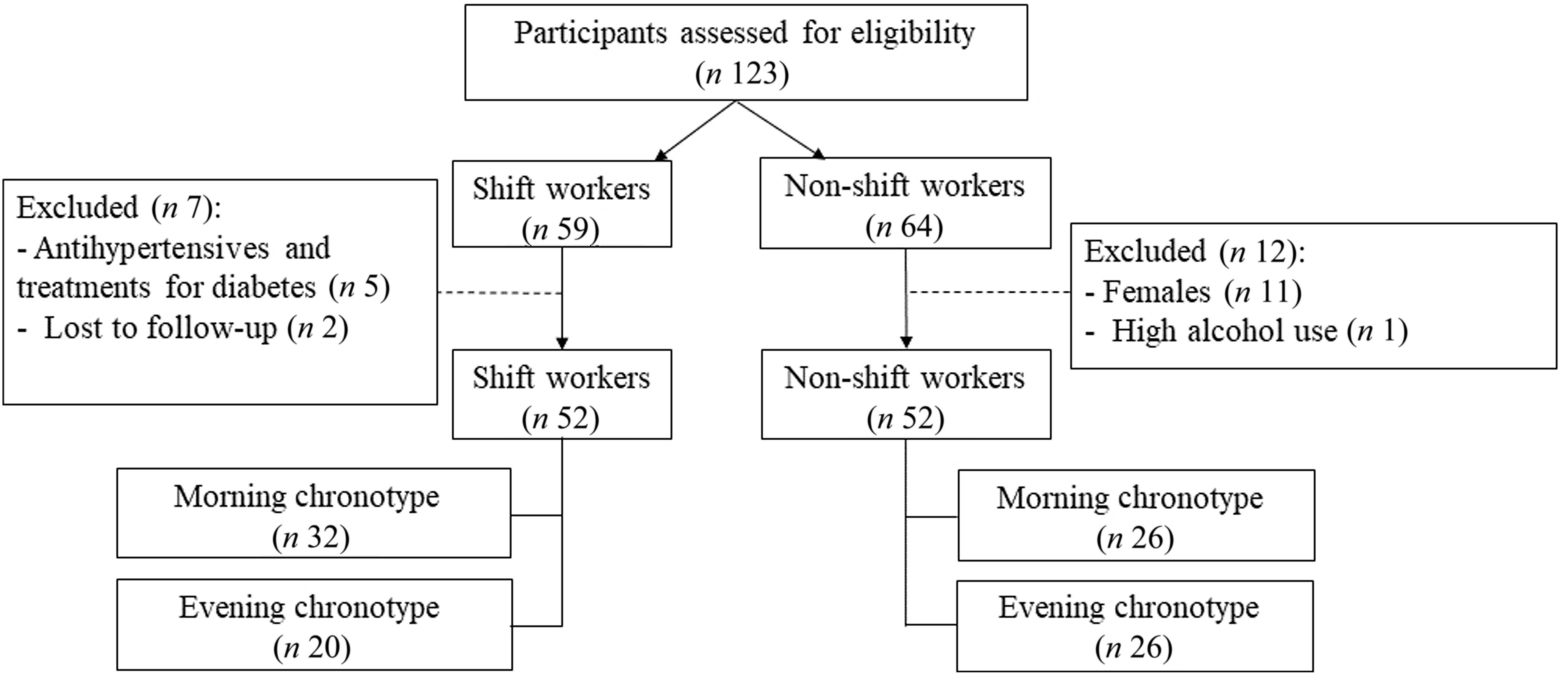
Participant recruitment and classification flow chart. This flow diagram illustrates the number of participants initially assessed, excluded and included in the final sample.

For this study, participants were security officers within the export processing zone in Sri Lanka to ensure job similarity between shift and non-shift groups. The nature of their work, including responsibilities such as guarding, surveillance and patrolling, was consistent across both shift and non-shift groups, ensuring minimal variability in physical job demands. The inclusion criterion was healthy male individuals aged between 30 and 60 years working full time on rotating shifts. Non-shift workers, comprising regular day-time workers, served as the reference group. Recruitment strategies involve various approaches such as posters, telephone calls, messages and notices that target workers from fire and security units. Shift workers were defined as those engaging in rotating shifts, alternating between day shifts (mainly between 07.30 and 16.00), night shifts (mainly between 23.00 and 07.45) and/or night shifts from 06.00–06.00 or 16.00–06.00. Conversely, non-shift workers were individuals not participating in rotating and night shifts in their work life. The exclusion criteria included females, individuals using statins, antihypertensives, or treatments for diabetes, those with higher smoking and alcoholic status, individuals with psychological disorders and participants who had lost their legs. Intermediate chronotypes were excluded before recruitment as per our predefined protocol. This decision was made to allow for clearer contrast in circadian alignment effects between biologically distinct phenotypes. Occupational physical activity levels were assessed using a structured questionnaire. Based on responses and classification criteria, all participants were classified as having a sedentary occupational lifestyle, characterised by prolonged standing or minimal movement, with no vigorous physical exertion. The presentation outlined the study’s objectives and extended invitations for participation without offering financial incentives. The participants had access to group nutrition education on the basis of the study’s findings. Among the 123 initially recruited participants, five were excluded because of the use of antihypertensives and treatments for diabetes, eleven were excluded because they were females, two were excluded because they were lost to follow-up and one was excluded because he had the highest alcoholic status. The main analysis included a total of 104 participants (fifty-two shift workers and fifty-two non-shift workers). Participant flow can be seen in Figure 1.

### Data collection

#### Shift work

Information about participants’ current shift work status (including work schedules and types of shifts), the frequency of (night) shifts (number of each shift type per month) and the duration of (night) shift work, as well as the total number of years of (night) shift work, was collected during the baseline and follow-up assessments. An interviewer-administered questionnaire was used to collect information from the workers. The interviews were conducted in person. The questionnaire comprehensively covered all major aspects of shift work.

#### Anthropometrics

Height was measured using a stadiometer to the nearest 0·5 cm. While participants were wearing light clothing, weight and BMI were determined by using the multi-frequency segmental body composition analyser Tanita MC-780 (Tanita) with the following settings: standard body type and –1 kg for clothing. Waist and hip circumferences were measured using a measuring tape following a standardised protocol that provided detailed instructions for accurate measurement. All measurements were taken before the start of the work shift, with participants in light clothing and no shoes. If there was a difference of more than one unit (in centimetres/kilograms) between the first two measurements, a third measurement was taken. The average of the three measurements was subsequently calculated. BMI was calculated as the ratio of weight (in kg) to the square of height (in m^2^).

#### Cardiometabolic risk markers

Blood samples (3·5 ml) were collected into serum separator tubes (BD vacutainer) from participants following a 12-h overnight fast (before the commencement of a day shift for both day and night workers) and left at room temperature for 30 min until centrifugation at 1700 × g for 15 min at 20°C to obtain serum which was stored at –20°C until analysis. These serum samples were used to determine lipids (total cholesterol, HDL-cholesterol and TAG) and glucose, with the use of an automated biochemistry analyser (Thermo Scientific, Indiko) with reagents from Thermo Scientific (Thermo Scientific). Fasting LDL-cholesterol was estimated by using the Friedewald formula^(14)^. Blood pressure was measured in triplicate by using an OMRON M6 automatic digital blood pressure monitor. A trained research student performed these measurements, ensuring that each measurement was taken at least 5 min apart, and the average of the three readings was used in the data analysis.

#### Dietary assessment

Dietary intake was assessed using a single 24-h dietary recall (representing both normal days and work days). Participants were provided with common guidance during group sessions at their workplace on how to provide dietary information. This guidance included instructions on measuring food quantities (using glasses, cups and spoons), specifying food preparation details (such as recipes, commercial brands and restaurant names) and recording meal starting times on research forms. Researchers estimated the amounts of food and beverages consumed based on the information provided by participants in household measurements, aided by photographs of common household utensils. Participants were questioned by researchers trained in food measurement units about their eating habits. All meal and snack intakes were reported along with their respective intake times. A trained dietitian reviewed the forms to ensure completeness and consistency.

#### Chronotype

The assessment of chronotype was conducted via the Munich Chronotype Questionnaire (MCTQ), which is a validated tool for assessing individual chronotypes that reflect preferences in circadian rhythms^(15)^. The chronotype was determined on the basis of participants’ reported mid-sleep time (*MSF*) on free days. In the questionnaire, the participants provided information regarding the time they spent before sleeping (sleep onset) and the time they woke up. Sleep duration was calculated as the difference between the reported sleep onset and awakening times. The *MSF* was computed via the following formula:

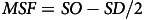




A higher *MSF* indicates an evening-oriented chronotype, whereas a lower *MSF* indicates a morning-oriented chronotype. Individuals who reported using an alarm clock on free days were excluded from the exploratory analysis, as their *MSF* values may be influenced by external factors and may not accurately reflect their internal circadian rhythm^(15)^. Intermediate chronotypes were excluded as part of the study’s design. Focusing on the two extreme chronotypes enhances biological contrast and statistical power when assessing vulnerability to shift-work-related circadian misalignment^(11,16)^.

### Statistical analysis

The statistical analysis aimed to explore associations, trends, and differences in the collected data, employing various tests and models to assess the study’s objectives. All analyses were performed via SPSS version 26.0. Descriptive statistics were calculated to present the basic features of the study variables. The means and standard deviations were reported for continuous variables, whereas frequencies and percentages were reported for categorical variables. Independent *t* tests were used to compare demographic characteristics between shift workers and non-shift workers. Multiple linear regression analyses, adjusting for potential confounding factors such as age and family history of CVD, were conducted to investigate associations between night work parameters and various cardiometabolic risk factors. Night work parameters included current work status, years of current shift work, hours of duration of current shift work and intensity of night work. Multiple linear regression analyses were also performed to examine cardiometabolic risk factors among current non-shift workers and shift workers, stratified by chronotype (morning-oriented and evening-oriented). The significance level for all analyses was set at *P* < 0·05.

#### Sample size

Sample size was calculated to detect a difference in cardiometabolic risk markers between shift and non-shift workers. A medium effect size was assumed (Cohen’s *d* = 0·5), based on previous studies^(11)^. The power calculation through G * Power indicated that a minimum of fifty-two participants in each group was required for 80 % power level and a significance level of *α* = 0·05. The study aimed to have a total sample size of 104 participants, with an allocation of fifty-two individuals in each group, based on an anticipated response rate of 75 %. This calculation ensures adequate statistical power to detect accurate significant differences in cardiometabolic risk markers between the two groups.

## Results

### Characteristics of the study population

The study included a total of 104 participants, shift workers (*n* 52) and non-shift workers (*n* 52). Table 1 elucidates distinct differences in demographic characteristics, physiological measures and dietary intake between shift workers and non-shift workers. Compared with non-shift workers, shift workers presented a mean age of 43·3 years (sd 10·2), whereas non-shift workers presented a mean age of 41·2 years (sd 9·8) (*P* = 0·451). In terms of physiological measures, shift workers presented a higher pulse rate with a mean of 78·7 beats per minute (sd 15·2) than non-shift workers with a mean of 72·3 beats per minute (sd 11·2) (*P*-value = 0·015). Body fat percentage revealed a significant difference, showing a higher mean percentage in shift workers (31·7 % (sd 41·6)) compared with non-shift workers (22·7 % (sd 5·8)), with a *P*-value of 0·031. Additionally, LDL-cholesterol level (*P* = 0·081) showed trends towards significance, suggesting potential differences between shift and non-shift workers. Dietary intake variables indicated minor distinctions between the two groups. The percentage of energy from added sugar was slightly higher in shift workers (6·76 % (sd 0·79)) compared with non-shift workers (5·98 % (sd 0·75)), with a significant *P*-value of 0·001.

**Table 1.** Characteristics of the study population (*n* 104)

	Shift workers (*n* 52)	sd	Non-shift workers (*n* 52)	sd	*P*
Age, years, mean	43·3	10·2	41·2	9·8	0·451
	*n*	%	*n*	%	
Duration of current shift (years) (%)					
1–5	23	44·23	13	25	0·112
5–10	11	21·15	15	28·84	
> 10	18	34·62	24	46·16	
	Mean	sd	Mean	sd	
Duration of night shift (hours)					
< 8	9	17·3	N/A	N/A	N/A
8–12	24	46·15			
12–24	19	36·55			
Night shift intensity/month					
< 15	29	55·76	N/A	N/A	N/A
15–24	23	44·24			
Family history of CVD					
Present	25	48·07	25	48·07	1
Not present	27	51·93	27	51·93	
	*n*	%	*n*	%	
Chronotype (%)					
Evening-oriented chronotype	20	38·46	26	50	0·321
Morning-oriented chronotype	32	61·54	26	50	
	Mean	sd	Mean	sd	
Anthropometry					
BMI (kg/m^2^)	25·4	3·89	24·4	4·18	0·813
Waist circumference (cm)	92·7	11·48	88·01	9·58	0·681
Hip circumference (cm)	96·31	8·21	93·82	7·32	0·804
Waist:hip ratio	0·96	0·08	0·94	0·07	0·701
Visceral fat level	12·21	4·18	9·4	4·2	0·943
Fat mass (g)	16·61	6·2	15·3	6·38	0·584
Fat-free mass (g)	51·79	7·01	49·51	7·89	0·752
Muscle mass (g)	49·1	6·58	47·57	6·01	0·751
Bone mass (g)	2·71	0·33	2·56	0·31	0·811
	*n*	%	*n*	%	
Body fat (%)	31·7	6·57	22·7	5·76	0·031^*^
	Mean	sd	Mean	sd	
Biochemical analysis					
Fasting glucose (mmol/l)	5·04	1·57	4·89	1·43	0·612
Total cholesterol (mmol/l)	5·6	1·08	5·45	0·93	0·317
TAG (mmol/l)	1·61	0·72	1·36	0·75	0·512
HDL-cholesterol (mmol/l)	1·43	0·37	1·58	0·73	0·121
LDL-cholesterol (mmol/l)	3·86	1·06	3·4	0·88	0·081
Clinical analysis					
Blood pressure (mmHg)					
Systolic	138·56	19·1	128·5	17·6	0·748
Diastolic	79·67	14·01	78·21	11·79	0·821
Pulse rate (bpm)	78·71	15·21	72·28	11·23	0·015^*^
Dietary intake					
Energy (kcal/d)	1978·71	77·2	1954·8	73·6	0·75
Protein (g/d)	60·31	3·48	58·01	3·32	0·382
Fat (g/d)	51·2	2·85	49·71	2·72	0·801
Carbohydrates (g/d)	313·47	13·68	313·01	13·01	0·69
Fibre (g/d)	12·24	0·57	11·91	0·54	0·538
Starch (g/d)	245·51	10·31	242·92	9·82	0·677
Total sugar (g/d)	43·28	4·2	41·51	4·01	0·747
Retinol equivs (µg/d)	246·82	49·22	277·64	46·9	0·878
Vitamin D (µg/d)	4·09	0·48	3·92	0·46	0·877
Vitamin E (mg/d)	6·37	6·45	1·34	6·15	0·16
%Energy from fat	23·9	0·88	24·01	0·84	0·3
%energy from protein	12·57	0·4	12·48	0·38	0·449
%Energy from carbohydrate	63·28	1·03	63·28	0·98	0·533
%Energy from saturated fat	7·53	0·6	7·46	0·63	0·406
%Energy from added sugar	6·76	0·79	5·98	0·75	0·001^*^

Data are presented as mean ( 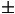 sd) for continuous variables, or *n* (%) for categorical variables, with *P*-values estimated by *t* tests and *χ* ^2^ tests, respectively.

* Significant at 0·05 significance level.

Several other variables included in the analysis had higher *P*-values, suggesting that there were no significant differences between the two groups. However, it is important to note that given the limitations of unadjusted analysis, the results based on independent *t* tests require further investigation to provide conclusive findings. Adjustments for potential confounders in subsequent analyses in Tables 2 and 3 may provide a more nuanced understanding of the observed differences.

**Table 2.** Adjusted mean differences in cardiometabolic risk factors, by shift work parameters

	*n*	Waist:hip	95 % CI	BMI (kg/m^2^)	95 % CI	SBP (mmHg)	95 % CI	DBP (mmHg)	95 % CI	Pulse rate	95 % CI	Visceral fat (g)	95 % CI	Body fat (%)	95 % CI	Fasting glucose (mmol/l)	95 % CI	TC (mmol/l)	95 % CI	TAG (mmol/l)	95 % CI	HDL-cholesterol (mmol/l)	95 % CI	LDL-cholesterol (mmol/l)	95 % CI
Current work status																									
Non-shift work	52	Ref		Ref		Ref		Ref		Ref		Ref		Ref		Ref		Ref		Ref		Ref		Ref	
Shift work	52	0·02^*^	–0·05, 0·01	1·01	–2·57, 0·55	10·1^*^	–17·2, −2·98	1·48	–6·52, 3·55	6·46^*^	–11·6, −1·26	2·84	–4·47, −1·21	9·0^*^	–20·5, 2·53	0·15	–0·43, 0·74	0·15	–0·55, 0·23	0·25^*^	–0·03, 0·54	0·15	–0·07, 0·38	0·35^*^	–0·75, 0·04
Years duration of current shift work (includes both shift and non-shift workers)																									
1–5 years	36	Ref		Ref		Ref		Ref		Ref		Ref		Ref		Ref		Ref		Ref		Ref		Ref	
5–10 years	26	0·05^*^	–0·09,–0·01	1·17	–3·24, 0·89	12·6^*^	–21·9, −3·15	3·02	–9·65, 3·60	5·38	–12·1, 1·46	2·89^*^	–5·06, −0·72	12·8	–27·9, 2·37	0·01	–0·8, 0·8	0·04	–0·48, 0·57	0·35	–0·04, 0·74	–0·14	–0·45, 0·16	0·11	–0·43, 0·66
> 10 years	42	–0·01	–0·02, 0·04	0·55	–2·37, 1·27	1·36	–9·67, 6·94	1·82	–7·67, 4·02	8·35^*^	–14·39, −2·38	–0·99	–1·81, 2·01	11·17	–24·5, 2·16	0·04	–0·68, 0·69	0·40^*^	–0·05, 0·86	0·34^*^	0·01, 0·68	0·06	–0·20, 0·32	0·27	–0·19, 0·74
Hours duration of current shift work																									
< 8 h	9	Ref		Ref		Ref		Ref		Ref		Ref		Ref		Ref		Ref		Ref		Ref		Ref	
8–12 h	24	0·05^*^	–0·94,–0·16	1·11	–3·24, 0·89	12·6^*^	–21·9, −3·15	3·02	–9·65, 3·60	5·33	–12·1, 1·46	2·89^*^	–5·06, −0·72	12·8	–27·9, 2·30	0·14	–0·83· 0·53	0·14	–0·61, 0·32	0·01	–0·33, 0·35	0·22	–0·48, 0·03	0·77	–0·39, 0·55
12–24 h	19	0·01	–0·26, 0·43	0·55	–2·35, 1·27	1·36^*^	–9·67, 6·94	1·82	–7·67, 4·02	8·38^*^	–14·4, −2·38	0·09^*^	–1·81, 2·01	11·2	–24·5, 2·16	0·45	–0·41, 1·32	0·17	–0·77, 0·41	0·21	0·64, 0·23	0·16	–0·49, 0·17	0·01	–0·59, 0·61
Intensity of night work																									
Non-shift work	52	Ref		Ref		Ref		Ref		Ref		Ref		Ref		Ref		Ref		Ref		Ref		Ref	
< 15 nights	29	0·02	–0·01, 0·06	1·31	–0·55, 3·17	9·88^*^	1·32, 18·4	2·04	–3·94, 8·04	6·37^*^	0·14, 12·6	3·71^*^	1·79, 5·62	15·2^*^	1·64, 28·8	0·11	–0·60, 0·82	0·19	–0·67, 0·28	0·32^*^	–0·68, 0·02	0·17	–0·45, 0·97	0·04	–0·44, 0·52
> 15 nights	23	0·01	–0·02, 0·05	0·46	–1·51, 2·44	8·96	–0·15, 18·1	–0·98	–7·32, 5·39	4·91	–1·72, 11·5	1·56	–0·47, 3·60	1·09	–13·34, 15·53	0·32	–1·07, 0·42	0·38	–0·11, 0·87	0·14	–0·51, 0·23	0·15	–0·44, 0·13	0·55^*^	0·05, 1·05

SBP, systolic blood pressure; DBP, diastolic blood pressure; TC, total cholesterol.

All analyses are adjusted for age and family history of CVD.

* *P* < 0·05.

**Table 3.** Adjusted mean differences in cardiometabolic risk factors among current shift workers, stratified by chronotype

	*n*	BMI (kg/m^2^)	95 % CI	SBP (mmHg)	95 % CI	DBP (mmHg)	95 % CI	Pulse rate	95 % CI	Visceral fat (g)	95 % CI	Body fat (%)	95 % CI	Fasting glucose (mmol/l)	95 % CI	TC (mmol/l)	95 % CI	TAG (mmol/l)	95 % CI	HDL-cholesterol (mmol/l)	95 % CI	LDL-cholesterol (mmol/l)	95 % CI
Current shift work status																							
Morning-oriented chronotype																							
Non-shift work	26	Ref		Ref		Ref		Ref		Ref		Ref		Ref		Ref		Ref		Ref		Ref	
Shift work	32	0·85	–2·81, 1·11	10·29	–20·19, 0·36	3·71	–9·98, 2·54	4·98	–12·31, 2·33	2·04	–4·19, 0·11	0·07	–2·57, 2·73	0·16	–0·67, 1·00	0·38	0·95, 0·18	0·25	–0·03, 0·53	0·07	–0·33, 0·18	0·35	–0·94, 0·23
Evening-oriented chronotype																							
Non-shift work	26	Ref		Ref		Ref		Ref		Ref		Ref		Ref		Ref		Ref		Ref		Ref	
Shift work	20	1·55	–4·14, 1·04	9·39^*^	–19·19, 0·33	1·97	–6·55, 10·55	9·44^*^	–16·49,–1·95	3·88^*^	–6·54,–1·27	13·00	–31·89, 5·89	0·05	–0·65, 0·76	0·10	–0·69, 0·48	0·13	–0·43, 0·69	–1·40	–0·07, 0·37	0·53^*^	–1·18, 0·05

SBP, systolic blood pressure; DBP, diastolic blood pressure; TC, total cholesterol.

All analyses are adjusted for age and family history of CVD.

* *P* < 0·05.

### Cardiometabolic risk factors by shift work parameters among shift workers and non-shift workers

The investigation delving into the association between night work parameters and cardiometabolic risk factors is presented in Table 2. In the comparison of current work status, where non-shift workers served as the reference group, notable disparities surfaced. Shift workers presented a significantly higher waist: hip by 0·02 cm (95 % CI −0·05, 0·01), elevated systolic blood pressure (SBP) by 10·10 mmHg (95 % CI −17·28, −2·98), an increased pulse rate by 6·46 (95 % CI −11·66, −1·26), an increased fasting TAG level by 0·25 mmol/l (%95 CI –0·03, 0·54), higher LDL-cholesterol level by 0·35 mmol/l (95 % CI −0·75, 0·04) and a higher body fat percentage by 9·0 % (95 % CI −20·5, 2·53). Exploring the impact of years of current shift work, significant mean differences emerged. For those engaged in 5–10 years of shift work, a notable increase in waist:hip ratio (0·05, 95 % CI −0·09, −0·01) and SBP (12·56 mmHg, 95 % CI −21·9, −3·15) was evident. Individuals with over 10 years of current shift work displayed a pronounced rise in pulse rate (8·38, 95 % CI −14·39, −2·38), fasting total cholesterol (0·40, 95 % CI −0·05, 0·86), fasting tTAG (0·34, 95 % CI 0·01, 0·68) and visceral fat (2·89 g, 95 % CI −5·06, −0·72).

In the examination of the duration of current shift work, significant mean differences were identified. Notably, individuals working 8–12 h manifested an increased waist:hip ratio (0·05, 95 % CI −0·94, −0·16) and SBP (12·56 mmHg, 95 % CI −21·98, −3·15). Those working 12–24 h demonstrated a marginal increase in SBP (1·36 mmHg, 95 % CI −9·67, 6·94) and a higher pulse rate (8·38, 95 % CI −14·39, −2·38). The visceral fat content also exhibited notable differences, with 8–12 h (2·89 g, 95 % CI −5·06, −0·72) and 12–24 h (0·09 g, 95 % CI −1·81, 2·01). Examining the intensity of night work, with non-shift work as the reference, significant mean differences surfaced. Those working fewer than fifteen nights showcased elevated SBP (9·88 mmHg, 95 % CI 1·32, 18·44), TAG (0·32, 95 % CI –0·68, 0·02), pulse rate (6·37, 95 % CI 0·14, 12·61), visceral fat content (3·71 g, 95 % CI 1·79, 5·62) and body fat percentage (15·21 %, 95 % CI 1·64, 28·77). Notably, individuals working more than fifteen nights manifested an increased LDL-cholesterol level (0·55, 95 % CI 0·05, 1·05).

### Cardiometabolic risk factors among current non-shift workers and shift workers, stratified by chronotype

The investigation of cardiometabolic risk factors among current non-shift workers and shift workers, stratified by chronotype, is shown in Table 3. The comprehensive array of selected cardiometabolic risk factors included BMI (kg/m^2^), SBP (mmHg), diastolic blood pressure (DBP) (mmHg), pulse rate, visceral fat (g), body fat analysis (%), fasting glucose (mmol/l), total cholesterol (mmol/l), TAG (mmol/l), HDL-cholesterol (mmol/l) and LDL-cholesterol (mmol/l). The analysis was divided into two distinct sections on the basis of chronotype to examine circadian rhythm patterns. Within each section, participants were further categorised into non-shift workers and shift workers, with non-shift workers considered as the reference group.

In the morning-oriented chronotype section, none of the cardiometabolic risk factors presented a *P*-value below the 0·05 significance threshold. Conversely, within the evening-oriented chronotype section, several notable mean differences were observed. Shift workers with an evening-oriented chronotype demonstrated a significant mean difference of 9·39 mmHg (95 % CI −19·19, 0·33) in SBP, 0·53 mmol/l (95 % CI −1·18, 0·05) in LDL-cholesterol, 9·44 (95 % CI −16·49, −1·95) in pulse rate and 3·88 g (95 % CI −6·54, −1·27) in visceral fat content were noted, indicating distinct cardiometabolic profiles among shift workers with an evening-oriented chronotype.

## Discussion

The research results presented provide a comprehensive overview of the complex relationships among demographic data, physiological parameters, dietary intake and cardiometabolic risk factors in the context of shift work. Thorough investigation of cardiometabolic risk factors in relation to various shift work parameters expands our understanding of the diverse effects of irregular work schedules on cardiovascular and metabolic health. The increased waist-to-hip ratio, SBP, pulse rate, TAG, LDL-cholesterol and body fat percentage in shift workers represent potential health risks that require comprehensive attention. When analysing the correlation between night shifts and cardiometabolic risk factors, our results are consistent with previous research indicating an association between current night work and increased cardiometabolic risk markers^(3)^. However, it is important to note that there is no complete uniformity across all studies. Discrepancies may be attributed to methodological differences, including possible over-adjustment of confounding factors, different definitions of night-shift work and the influence of selection bias^(17)^.

The recognised increased waist-to-hip ratio in shift workers implies severe consequences for cardiovascular health, as an increased ratio is often linked to an increased risk of CVD. A cross-sectional study of female hospital employees established that those engaged in rotating shift systems exhibited an increased waist circumference compared with their non-shift working counterparts^(18)^. Shift workers exhibit significantly elevated SBP and pulse rate compared with non-shift workers. The elevated pulse rate might indicate increased activity of the sympathetic nervous system, which reflects the physiological stressors due to irregular working schedules^(19)^. Blood pressure levels tend to rise in periods of wakefulness, especially during work shifts, in contrast to non-working hours. Considering that working hours often coincide with the night-time, a period during which blood pressure is normally supposed to fall, engagement in shift work, particularly at night, could disrupt the normal pattern of blood pressure, leading to the risk of CVD^(20)^. A variety of epidemiological studies have continually shown higher blood pressure levels in shift workers compared with those working regular daytime shifts^(19)^.

Our analysis of fasting lipid profiles, which includes parameters such as total cholesterol, TAG and LDL-cholesterol, showed some interesting trends among the shift workers. Despite changes in the overall levels of total cholesterol not being statistically significant, the trends in the levels of LDL-cholesterol suggest potential implications in cardiovascular risk. Shift workers revealed a tendency towards higher LDL-cholesterol levels compared to non-shift workers. High levels of LDL-cholesterol have often been associated with an increased risk of CVD^(3,10,13)^. Moreover, increased body fat percentage leads to questions about the metabolic consequences of circadian disruptions. In some studies, overweight (OR: 1·44; 95 % CI 1·06, 1·95) and higher BMI (*β*: 0·56 kg m^−2^, 95 % CI 0·10, 1·03) can be observed in shift workers, compared with day workers^(3)^. Further research on the long-term health effects of these demographic and physiological differences is warranted to appropriately develop health interventions for those with prolonged exposure to shift work.

The significantly large increment in SBP among shift workers with 5–10 years of working underscores the cumulative nature of cardiovascular risk associated with long-term exposure to non-normal working hours. Similarly, one study shows that those who had a history of night work for 10 or more years also showed a trend towards larger differences in cardiometabolic risk factors^(3)^. Knowing the temporal dynamics of these changes and modifiable factors would help design targeted interventions. The steep increase in pulse rate among those with more than 10 years of current shift work raises questions about the adaptability of the cardiovascular system to prolonged exposure to irregular work schedules. Investigating whether this is a transient response or a sustained adaptation will provide insights into the long-term consequences of shift work on cardiovascular function. The study shows significant differences in total cholesterol levels among individuals with more than 10 years of current shift work. Elevated TAG and visceral fat levels were observed among shift workers, particularly those with more than 10 years of current shift work, and this may indicate that night work parameters could be related to lipid metabolism. However, few cardiometabolic markers were worse among shift workers with 5–10 years of experience compared with those with both shorter and longer durations, suggesting a potential non-linear relationship. One explanation is a ‘burnout’ effect, where cumulative stress and circadian misalignment may peak in the mid-term. Another possibility is a ‘healthy worker effect’, where individuals unable to physiologically tolerate long-term shift work may leave such roles earlier, resulting in a selection bias among long-term workers. Future longitudinal studies are needed to examine these patterns more robustly.

Moreover, this study of visceral fat content in relation to various shift work parameters helps to understand the potential risk factors of metabolic disorders. The differences in visceral fat and SBP between those working 8–12 h and 12–24 h of current shift work are notable, indicating the influence of work duration on metabolic outcomes. Longitudinal studies, following changes in SBP and visceral fat over time, coupled with serial dietary assessment and physical activity measurements, will add much-needed detail to the understanding of the metabolic implications of irregular work schedules.

The higher levels of LDL-cholesterol in people who work at night more than fifteen nights per month suggest a potential dose–response relationship between the frequency of night work and adverse lipid profiles. Few studies have explored the length and frequency of night shifts and how these are associated with cardiometabolic risk factors, and this heterogeneity makes direct comparisons difficult^(3)^. Also, it remains unclear why perpetual night work frequency is associated with higher levels of SBP, pulse rate, visceral fat, percentage body fat and TAG levels^(11,21,22)^. Notably, a recent study suggested that there is no clear association between a history of night work or the frequency of night shifts and cardiometabolic risk factors^(11)^. On the other hand, it has been observed by a longitudinal study that more years of night-shift work were associated with an increased risk of CVD^(13)^.

Stratifying the analysis by chronotype adds a layer of complexity to our understanding of the interplay between circadian rhythms and shift work. Morning-oriented types did not differ significantly in cardiometabolic risk factors, whether they worked non-shift or shift work. In the evening-oriented chronotype category, however, some significant differences in mean values emerged, especially in the shift worker group. Notable mean differences in SBP, LDL-cholesterol, pulse rate and visceral fat content further underlined the distinct cardiometabolic profiles associated with evening-oriented shift workers. Recent studies also suggest that for shift workers, an evening chronotype is related to a higher risk of obesity^(11)^. Moreover, some findings from studies not in the setting of shift work point out that having an evening chronotype may be associated with a higher likelihood of cardiometabolic diseases^(16,23)^. Notably, a large difference in BMI between day and shift workers was particularly pronounced among those with evening chronotypes (*β*: 0·97 kg m^–2^, 95 % CI 0·21, 1·73), while no such difference was found among those with morning chronotypes (*β*: 0·04 kg m^–2^, 95 % CI –0·85, 0·93)^(3)^.

The circadian system is regulated by a primary pacemaker controlling the sleep–wake cycle and the production of melatonin in the pineal gland. This pacemaker prompts the pineal gland to release melatonin, a crucial marker of the circadian rhythm, particularly during the dark phase of the light–dark cycle^(24)^. Exposure to light at night directly impacts this process. Shift workers are frequently exposed to artificial light during biological night, a time when the human body anticipates darkness and melatonin secretion normally peaks. The nocturnal light exposure suppresses melatonin production, shifts the phase of the circadian rhythm and influences sleep control and metabolic function^(25)^. Furthermore, shift workers have considerably higher cortisol levels in daytime sleep than in the night-time sleep of people with standard daytime schedules^(26)^. Additionally, during night-time wakefulness, cortisol levels are lower in shift workers compared with daytime workers when they are awake^(27)^. Melatonin influences blood pressure by affecting endothelial cells^(28,29)^. Furthermore, melatonin treatment has been shown to reduce both systolic and DBP^(28)^. These effects of continuous melatonin intake on blood pressure are necessarily time-dependent^(28)^. Consequently, irregular light exposure and additional disturbance in melatonin secretion resulted in blood pressure dysregulation^(30)^. Moreover, evening chronotypes, who naturally get their first light exposure of the day later in the day or in the night, may be more prone to this perturbation, particularly if they work rotating or night shifts^(10)^. Recent research has determined that late-phase light exposure is linked to elevated levels of LDL-cholesterol and TAG, particularly in individuals with evening types^(31,32)^. These mechanisms may be the cause of the significantly higher lipid and blood pressure values in this subgroup in our study. The relationship of increased TAG, total cholesterol and LDL–C levels in certain subgroups of shift workers points towards a complex interplay between the nature of night work and lipid metabolism. This complex interplay of these associations is probably influenced by a variety of lifestyle factors, disturbances in circadian rhythms and physiological stressors. Further research is needed to identify the underlying mechanisms that give rise to the observed lipid-related differences and to develop targeted interventions that address the specific problems faced by shift workers.

Further research is needed to understand the mechanisms by which chronotype, shift work and cardiometabolic outcomes are linked in order to develop effective targeted interventions. Chronotype-based intervention, for instance, personalised sleep hygiene recommendations, time-restricted feeding or optimised work schedules, could be used to try to minimise the health risks associated with shift work. It is also imperative to find out whether cardiometabolic profiles in evening-oriented chronotype shift workers are reversible or open to intervention.

When interpreting the results of this study, several limitations must be considered. Sample size may limit the statistical power and generalisability of findings. While the study intentionally focused on morning and evening chronotypes to enhance biological contrast, the exclusion of intermediate chronotypes may limit the generalisability of the findings to the broader working population, where intermediate types are more prevalent. Expanding the sample size could have strengthened the observed associations with shift work, chronotype and cardiometabolic risk markers. Efforts were made to control collinearity by performing the variance inflation factor analysis, but some degree of residual multicollinearity may not be completely avoided. Some confounding factors, such as stress and quality of sleep, may have impacted findings. Although sleep quality and total duration were not assessed, sleep timing was captured via MCTQ to accurately determine chronotype. Although the MCTQ includes an item assessing habitual time spent outdoors, participant responses were inconsistent and incomplete. As a result, this variable was excluded from the final analysis. Future research should consider using objective measures of light exposure, such as actigraphy or wearable light sensors, to provide more precise data on photic input. Additionally, since this is a case–control study, some risk factors may have changed by the time of data collection due to participants’ awareness of their condition. This may have influenced the true relationship of certain current factors.

In conclusion, shift workers had higher metabolic risk markers than non-shift workers. Shift workers with evening chronotypes had higher cardiometabolic risk than morning chronotype shift workers. Future research should focus on developing preventive measures for shift workers.
